# Changes in Acylglycerols Composition, Quality Characteristics and *In vivo* Effects of Dietary Pumpkin Seed Oil upon Thermal Oxidation

**DOI:** 10.3389/fchem.2017.00055

**Published:** 2017-07-26

**Authors:** Alam Zeb, Sultan Ahmad

**Affiliations:** Laboratory of Biochemistry, Department of Biotechnology, University of Malakand Lower Dir, Pakistan

**Keywords:** pumpkin seed oil, rabbits, oxidized lipids, acylglycerols, toxicity, hematology, histology

## Abstract

This study was aimed to determine the acylglycerols composition, quality characteristics, and protective role of dietary pumpkin seed oil (PSO) in rabbits. PSO was thermally oxidized and analyzed for quality characteristics and acylglycerols composition using reversed phase high performance liquid chromatography with diode array detection (HPLC-DAD). Oxidized and un-oxidized oil samples were fed to the rabbits in different doses for 2 weeks. The changes in the serum biochemistry, hematology, and liver histology were studied. The levels of quality parameters such peroxide value (PV), anisidine value (AV), total phenolic contents (TPC), thiobarbituric acid reactive substances (TBARS), conjugated dienes (CD) and conjugated trienes (CT) significantly increased with thermal treatment. HPLC analyses revealed 10 individual triacylglycerols (TAGs), total di-acylglycerols (DAGs), mono-acylglycerols (MAGs), and total oxidized TAGs. Trilinolein (LLL), 1-oleoyl-2,3-dilinolinoyl glycerol (OLL), triolein (OOO) and 1,2-distearoyl-3-palmitoyl glycerol (SSP) were present in higher amounts and decreased with thermal treatment. Animal's studies showed that oxidized oils decreased the whole body weight, which was ameliorated by the co-administration of un-oxidized oils. The levels of serum biochemical parameters were improved by co-administration of pumpkin seed oils. There were no significant effects of both oxidized and un-oxidized PSO on the hematological and histological parameters of rabbits. In conclusion, nutritionally important triacylglycerols were present in PSO with protective role against the toxicity of its corresponding oxidized oils.

## Introduction

Pumpkin belongs to the cucurbitacae family is widely known as palatable and tasty food. Pumpkin seeds are used as a useful source of edible oil with several beneficial properties. Pumpkin seed oil (PSO) had been observed to decrease free radicals and was helpful against arthritis in rats (Fahim et al., 1995). The oil is rich in phytoestrogens, upon supplementation to rats inhibit changes in the plasma lipids and cardio-vascular outcomes (Gossell-Williams et al., 2008). In hypercholesterolemic rats, the PSO administration showed a strong anti-atherogenic and hepato-protective effects (Makni et al., 2008). PSO was found helpful against rapid cell growth in breast and colon cancer (Medjakovic et al., 2016) and against wound healing in animals (Bardaa et al., 2016). Yasir et al. (2016) showed that pumpkin seed extract had a high antioxidant and geno-protective activity and thus was highly beneficial as a food supplement. PSO is rich in several bioactive compounds such as, tocopherols, phytosterols (Rabrenović et al., 2014), carotenoids (Seo et al., 2005), and phenolic compounds (Nyam et al., 2009). The oil is rich in oleic acid and linoleic acid (Nawirska-Olszańska et al., 2013). Among the triacylglycerols (TAGs), di-linoleoyl stearin (LLS), di-linoleoyl palmitin (LLPa), di-linoleoyl olein (LLO), and trilinolein (LLL) were the major TAGs, which showed significant variations during ripening of seed (Deineka et al., 2003; Nederal et al., 2012), and play important role in stability of the oil. Tocopherol and squalene also contributed to the stability of PSO (Naziri et al., 2016).

Thermal treatment is one of the common practice in preparation of foods from the kitchen to food industries and thus evaluation of frying oil is important for food chemist. It has been observed that frying produces primary oxidation products, which enter the food matrix and are thus consumed by humans. These primary oxidation compounds are formed from TAGs present in oil or frying medium (Zeb, 2015). Murkovic et al. (2004) reported that significant variations occurred in the linoleic acid and tocopherol contents during roasting of pumpkin seeds. Similarly, roasting significantly decreased the amount of equivalent carbon number (ECN)-54 TAGs than other TAGs in pumpkin seed of different varieties (Yoshida et al., 2006). These authors reported that palmitoyl di-olein (POO), di-palmitoyl linolein (PPL), triolein (OOO), palmitoyl oleoyl linolein (POL), stearoyl oleoyl linolein (SOL), di-oleoyl linolein (OOL), palmitoyl di-olein (PLL), oleoyl di-linolein (OLL) were present as major TAGs and varied significantly during roasting of pumpkin seeds. A recent study suggested that hydrolysates prepared from processing of pumpkin oil might help as alternative sources of dietary proteins with good nutritional quality, and protection against oxidative damage (Venuste et al., 2013). However, there is a lack of information about the food uses of fried or heated PSO in animal models and *in vivo* effects upon ingestion. The important medicinal and quality characteristics of the PSO has warranted us to determine for the first time the effects of thermal treatment on the chemical characteristics of PSO such as, acylglycerols composition and quality parameters as well as its co-administration in to the experimental animals.

## Materials and methods

### Materials

Fresh PSO was obtained from the local market in Lahore, Pakistan. Standard triolein (Pubchem CID, 5497163), trilinolein (Pubchem CID, 5322095), tristearin (Pubchem CID, 11146), tridecanoin (Pubchem CID, 69310), dioleoyl palmitin (Pubchem CID, 13183955), and tripalmitin (Pubchem CID, 11147), methanol (Pubchem CID, 887), were obtained from Sigma-Aldrich (Germany). 2-Propanol (Pubchem CID, 3776), was from Daejung Chemicals (South Korea). Fatty acids such as oleic acid (Pubchem CID, 445639), linoleic acid (Pubchem CID, 5280450) and linolenic acid (Pubchem CID, 5280934), were from TCI (Tokyo Chemical Industries, Japan). Gallic acid (Pubchem CID, 370) was from BDH (BDH, England). Deionized double distilled water was obtained using Labtech (South Korea). All chemicals or reagents were of the HPLC grade with highest purity.

### Thermal oxidation

PSO (500 mL) was taken in open air hot plate. The conditions of thermal oxidation were temperature of 160°C, duration of continuous 9 h with surface to volume ratio of 0.04 cm^−1^ and under day light in a reaction chamber to mimic food frying in the daily life. The oil sample was stored in the refrigerator at −20°C. Using mass spectrometry, previous findings (Zeb, 2012) showed that there were no changes in the oxidation status, when oxidized oils are stored in a refrigerator at −20°C. The un-oxidized pumpkin oil was used as a control.

### Analyses of quality parameters

Both oxidized and un-oxidized PSO was analyzed for standard quality parameters. Peroxide and anisidine values were determined using AOCS methods (Firestone, 1998). Thiobarbituric acid reactive substances (TBARS) were measured using our recently reported method and were expressed as μmol/g (Zeb and Ullah, 2016). Conjugated dienes (CD) and trienes (CT) were measured as in terms of specific extinctions at 230 and 270 nm. The oil samples were diluted using *iso*-octane as solvent (Firestone, 1998). Total phenolic contents (TPC) were measured using Folin-Ciocalteu reagent at 765 nm based on standard gallic acid calibration curve in the concentration range of 5–100 mg/100 mL (y = 0.0094x + 0.0086, *R*^2^ = 0.9976) and expressed as mg/g of oil with details as reported recently (Zeb and Nisar, 2017).

### HPLC analyses of triacylglycerols

Oxidized and un-oxidized pumpkin seed oils were weighted (20 mg each) in 10 mL vials and dissolved in 2 mL isopropanol. The mixture was thoroughly vortexed for 5 min at 20°C in the absence of light. The mixture was filtered using 0.45 μm PFTE filter (Agilent Technologies, Germany) in to 2 mL HPLC vials. The samples were analyzed for acylglycerols composition using HPLC with diode array detection at 210 nm (Zeb and Murkovic, 2010). The HPLC system (Agilent Infinity Better 1260, Germany) was freshly calibrated with the standard TAGs and isocratic elution of 2-propanol in methanol (18%) was carried at 1 mL/min of flow rate. The separation was carried using Agilent rapid resolution C18 column (Agilent Technologies, Germany) with the specification of 4.6 × 100 mm with particle size of 3.5 μm and was maintained at 25°C. TAGs, DAGs, MAGs were determined with reference to the standard compounds or using ECN values. The standard calibration curves were prepared for LLL (5–50 mg/mL), OOO (5–30 mg/mL), SSP (1–20 mg/mL), and SSS (1–10 mg/mL), while the concentrations of other TAGs were determined using ECN values and the above calibration curves. DAGs were the mixture of LL, OO, OS, OP, SP, and SS in the concentration range of 5–20 mg/mL, while MAGs were mixture of mono-linoleoyl glycerol, mono-oleoyl glycerol and mono-stearoyl glycerol in the concentration range of 5–30 mg/mL. The TAGOX (1–20 mg/mL) were prepared by thermally oxidizing OOO using the procedure reported previously (Berner and Dieffenbacher, 1999).

### Animals feeding

Male rabbits with average weight of 1.3 ± 0.12 kg of the Himalayan strain were selected as the experimental animal for the current work. Thirty rabbits were obtained and acclimatized in the animal house of the University of Malakand for 2 weeks. The rabbits were housed in standard cages within the animal house in a light-controlled room (12 h dark/12 h light cycle) at 25°C and were given a standard pellet diet and water *ad libitum*. The experiments were approved initially by the Graduate studies committee of the Department of Biotechnology, for the proper care and experimentation of the animals and were finally approved by the advanced study and research board (ASRB) of the University of Malakand in its 18th meeting. The changes in the weight of the rabbits were measured by Marino digital balance (Marino, Japan).

Pumpkin seed oils were fed to the rabbits using oral gavage after diet. The rabbits were classified randomly into 10 groups containing minimum acceptable three replicates i.e., each group containing three rabbits for a period of 2 weeks with following details: Group 1 (NPO1) was fed on un-oxidized PSO with 1 g/Kg body weight. Group 2 (NPO2) fed un-oxidized PSO with 2 g/Kg body weight. Group 3 (NPO3) fed on un-oxidized PSO with 3 g/Kg body weight. Group 4 (OPO1) fed on oxidized PSO with 1 g/Kg body weight. Group 5 (OPO2) fed on oxidized PSO with 2 g/Kg body weight. Group 6 (OPO3) fed on oxidized PSO with 3 g/Kg body weight. Group 7 (ONPO1) fed on oxidized PSO with 3 g/Kg body weight and un-oxidized pumpkin seed oil with 1 g/Kg body. Group 8 (ONPO2) fed on oxidized PSO with 3 g/Kg body weight and un-oxidized PSO with 2 g/Kg body. Group 9 (ONPO3) was fed on oxidized PSO with 3 g/Kg body weight and non-oxidized PSO with 3 g/Kg body, while group 10 was control fed with normal diet without any oil supplementation.

### Analysis of biochemical parameters

After the completion of 1 week feeding blood sample were taken from the jugular vein of the rabbits for hematological and serum biochemical analysis. On completion of feeding scheme (2 weeks) all rabbits including control were anesthetized and slaughtered as per the detail reported procedure (Calasans-Maia et al., 2009). Blood samples were taken from all groups for the evaluations of biochemical and hematological parameters. Serum biochemical analyses include total cholesterol, triglycerides, HDL, LDL, glucose, and ALT were performed using standard reagent kits (Human Diagnostic, Germany) in triplicates.

### Hematology

For hematology fresh blood (2 mL) was collected in EDTA tubes. Complete blood hematology was done by automatic digital machine (NIHON CODEN, Japan) for the analysis of all essential parameters such as white blood cells (WBC), red blood cells (RBC), hemoglobin (Hb) and platelets.

### Liver histopathology

Liver sections were collected in formalin upon removal from the rabbit's body. Thin slides were prepared and stained as per the standard procedure reported (Zeb and Rahman, 2017). The picture was documented and analyzed for the any histological changes produced by the feeding of oxidized and un-oxidized pumpkin oils.

### Data analyses

All experiments were carried out in triplicate. Data obtained was analyzed for statistical variations using GraphPad prism software version 7.01 (GraphPad Software, Inc., 2016) with post hoc test of Tuckey's multiple comparison. Significant differences between mean values were determined at different *p*-values and represented by different letters where appropriate.

## Results

### Characteristics of pumpkin oils

**Figure 1:**
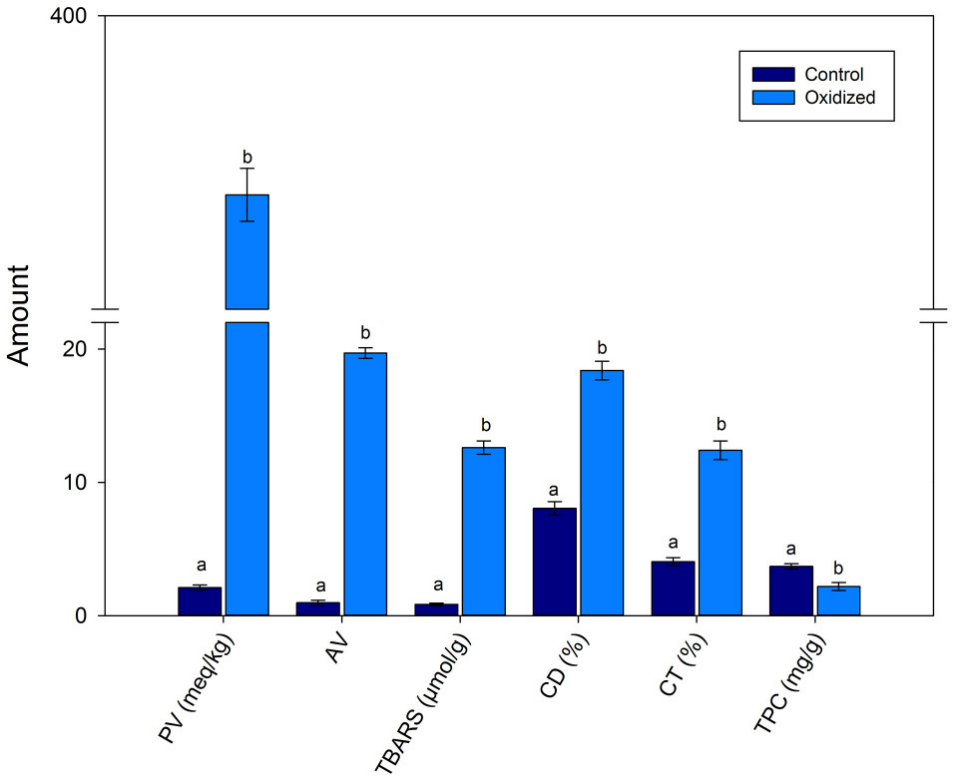
Effects of thermal treatment on the quality characteristics of pumpkin seed oil. Data are means with error bars of standard deviation (*SD*) of *n* = 3 per group. Different letters (a-b) in the same parameter represent significance at different *p*-values. PV is peroxide values (^*^); TBARS, thiobarbituric acid reactive substances (^**^); CD, conjugated dienes (^*^); CT, conjugated trienes (^**^); TPC, total phenolic contents (^***^), where ^*^p < 0.05, ^**^0.01, and ^***^0.001.

Figure 1 shows that PV was 2.1 and 375.6 meq/kg of un-oxidized pumpkin and oxidized pumpkin seed oil. This showed that a significant (*p* < 0.01) increased occurred in the PV with heating. Similarly, AV also increased from 0.96 to 19.6 of control and oxidized oil. The TBARS values increased from 0.84 to 12.6 μmol/g upon heating for 9 h. The CD and CT also increased with heating time confirming lipid oxidation. The TPC decreased with heating from 3.69 to 2.18 mg/g in control and oxidized PSO sample, respectively.

### Acylglycerols composition

**Figure 2:**
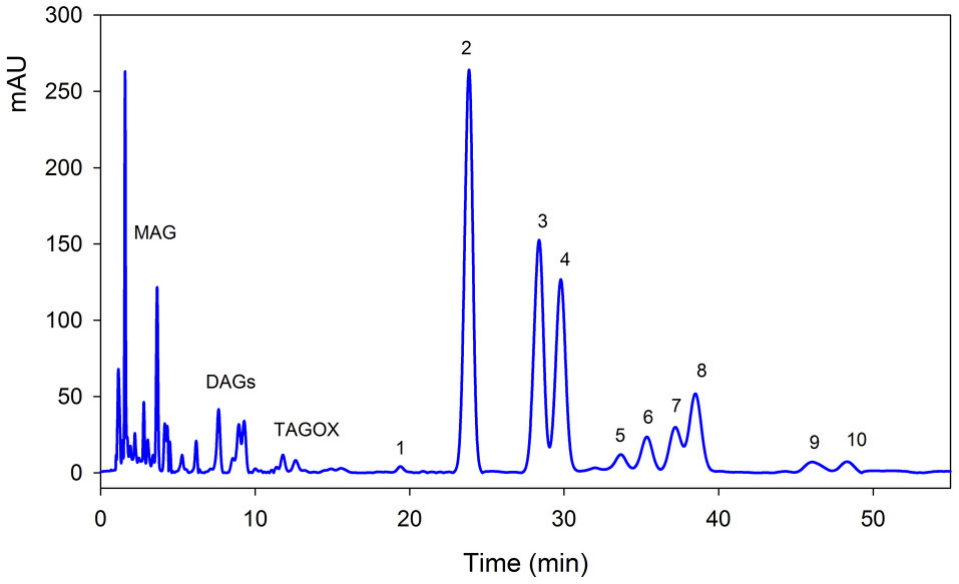
Typical HPLC-DAD chromatogram of pumpkin seed oil at 210 nm. The details of each peak is given in Table 1. MAG, mono-acylglycerols; DAGs, di-acylglycerols; TAGOX, oxidized triacylglycerols.

Triacylglycerols (TAGs) were separated using C18 column along with mono-acylglycerols (MAGs), di-acylglycerols (DAGs) and oxidized TAGs (TAGOX) as shown in the Figure 2. The amounts of TAGs, DAGs, MAGs and TAGOXs were 76.86, 7.57, 10.22, and 1.65 g/100 g, respectively, in control oil and changed to 71.55, 9.68, 12.8, and 4.51 g/100 g in oxidized samples. Ten triacylglycerols were identified as 1-linolenoyl-2,3-dilinoleoyl glycerol (LLLn), trilinolein (LLL), 1-oleoyl-2,3-dilinoleoyl glycerol (OLL), triolein (OOO), 1-palmitoyl-2,3-dioleoyl glycerol (POO), 2-oleoyl-1,3-dipalmitin (POP), 1-steroyl-2,3-dioleoyl glycerol (SOO), 3-palmitoyl-1,2-disteroyl glycerol (SSP), 2-oleoyl-1,3-disteroyl glycerol (SOS) and tristearin (SSS). The PSO was rich in LLL (26.4 g/100 g), OLL (17.4 g/100 g), OOO (14.7 g/100 g) and SSP (7.32/100 g) as shown in Table 1. Significant decrease in the amounts of the unsaturated TAGs upon heat treatment was observed. There was a small increase in the amount of saturated TAGs (SOO & SOS) with heating.

**Table 1:** Effect of thermal oxidation on the acylglycerols composition of pumpkin oil.

**Peak**	**Retention Time (min)**	**Triacylglycerol**	**Composition (g/100 g of oil)**	
			**Control oil**	**Oxidized oil**
1	19.3	LLLn	0.33 ± 0.01a	0.25 ± 0.05b
2	23.8	LLL	26.4 ± 0.5a	22.6 ± 0.7b
3	28.3	OLL	17.4 ± 0.5a	15.2 ± 0.3b
4	29.7	OOO	14.7 ± 0.5a	15.0 ± 0.5a
5	33.6	POO	1.22 ± 0.1a	0.99 ± 0.2a
6	35.3	POP	2.82 ± 0.04a	2.86 ± 0.05a
7	37.1	SOO	3.82 ± 0.01a	4.25 ± 0.03b
8	38.4	SSP	7.32 ± 0.02a	7.12 ± 0.01b
9	46.0	SOS	1.44 ± 0.01a	1.71 ± 0.01b
10	48.3	SSS	1.41 ± 0.1a	1.57 ± 0.2a
Total TAGs			76.86 ± 0.5	71.55 ± 0.6
Total DAG			7.57 ± 0.3	9.68 ± 0.3
Total MAG			10.22 ± 0.3	12.8 ± 0.5
Total TAGOX			1.65 ± 0.05	4.51 ± 0.1

*Values are mean of triplicate readings. Different letters (a-b) represent significant difference at p < 0.01 (Tuckey test). Quantification is based on the availability of standard calibration curves prepared from standard compounds. The identification of MAGs & DAGs are based on the standard mixture of compounds. TAG, triacylglycerols; DAG, di-acylglycerols; MAG, mono-acylglycerols and TAGOX oxidized TAGs. 3-linolenoyl-1,2-dilinoleoyl glycerol (LLLn), trilinolein (LLL), 1-oleoyl-2,3-dilinoleoyl glycerol (OLL), triolein (OOO), 1-palmitoyl-2,3-dioleoyl glycerol (POO), 2-oleoyl-1,3-dipalmitin (POP), 1-steroyl-2,3-dioleoyl glycerol (SOO), 3-palmitoyl-1,2-disteroyl glycerol (SSP), 2-oleoyl-1,3-disteroyl glycerol (SOS) and tristearin (SSS)*.

### Weight changes in rabbits

**Figure 3:**
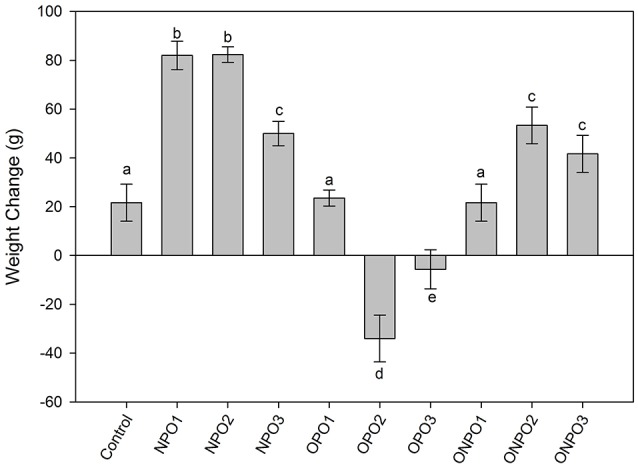
Effects of supplementation of oxidized and un-oxidized pumpkin seed oils on the relative changes in the body weight of the rabbits. NPO, none oxidized pumpkin oil; OPO, oxidized pumpkin oil; ONPO, oxidized and none-oxidized pumpkin combined. Numeral 1–3 represents the amount given. Different letters (a–e) represent significant at *p* < 0.05 using Tuckey test.

Supplementation of un-oxidized PSO significantly (*p* < 0.05) increased in the body weight of the rabbits, while a significant (*p* < 0.05) decrease in body weight was observed in OPO2 and OPO3 groups (Figure 3). Supplementation of oxidized pumpkin oil with 1 g/kg produced no significant effects on the body weight. However, an increased in the amount of oxidized oil produced significant (*p* < 0.05) increase in the body weight as compared to the control. The co-administration of un-oxidized oil with oxidized significantly improved the body weight.

### Serum biochemistry

Serum biochemical parameters were studied after 7 and 14 days of feeding of pumpkin seed oils. Table 2 shows that supplementation of un-oxidized PSO alone increased the levels of total cholesterols (TC) from control values of 66.3 and 69.3 mg/dL of 7 and 14 days of treatment. A significant (*p* < 0.05) decrease occurred from 89.3 to 79.3 mg/dL and 95.6 to 84.3 mg/dL with an increase in the dose rates (1–3 mg/kg) of un-oxidized oils during 7 and 14 days of treatment. Inversely, the oxidized oil significantly (*p* < 0.05) increased the total cholesterol in a dose dependent manner to a maximum of 93.6 and 89.7 mg/dL in 7 and 14 days, respectively. The co-administration of un-oxidized oils along with oxidized oils tends to decrease the amount of serum cholesterol with increase in the dose rate of un-oxidized pumpkin oils. However, the amounts of serum cholesterol were close to the values in OPO groups. Similar observations were also found in the case of serum triglycerides (TGs). The TGs in the control groups were 66.4 and 67.3 mg/dL, which increased significantly (*p* < 0.05) to 89.3 and 95.7 mg/dL in the NPO1 group. In OPO groups, significant (*p* < 0.05) increased occurred in the TGs values during 7 and 14 days. The co-administration of both oils revealed a significant (*p* < 0.05) increase of TGs in ONPO1 groups, which showed decline with an increase in the dose of un-oxidized oils. No significant changes were observed in the HDL-c levels in NPO groups during 7 days, while a significant (*p* < 0.05) occurred during 14 days of treatment. There was a significant (*p* < 0.05) decline in the HDL values in OPO groups, while co-administration of both oils significantly improved its values. Serum LDL values increased significantly (*p* < 0.05) in NPO groups, however, there was a dose dependent decline. In OPO groups, there was a significant (*p* < 0.05) increase in the LDL values with an increase in the amount of oxidized oils. The co-administration of both oils revealed a significant (*p* < 0.05) increase in the LDL values, which was found to decline in a dose dependent manner suggesting beneficial properties.

**Table 2:** Effects of oxidized and un-oxidized pumpkin seed oil supplementation on the serum biochemical parameters of rabbits.

**Group**	**Cholesterol (mg/dL)**		**Triglycerides (mg/dL)**		**HDL-cholesterol (mg/dL)**		**LDL-cholesterol (mg/dL)**		**Glucose (mg/dL)**		**ALT (U)**	
	**7**	**14**	**7**	**14**	**7**	**14**	**7**	**14**	**7**	**14**	**7**	**14**
Control	66.3 ± 2.3a	69.3 ± 1.6a	66.4 ± 2.3a	67.3 ± 3.0a	70.6 ± 0.7a	71.0 ± 1.0a	23.3 ± 1.5a	23.3 ± 1.1a	72.3 ± 1.2a	71.3 ± 1.5a	29.3 ± 0.5a	32.7 ± 1.0a
NPO1	89.3 ± 2.3b	95.6 ± 2.5b	89.3 ± 3.3b	95.7 ± 2.5b	72.0 ± 0.8a	75.3 ± 0.5b	47.2 ± 1.3b	44.2 ± 1.3b	60.0 ± 1.5b	53.0 ± 2.0b	33.3 ± 0.7b	33.3 ± 1.0a
NPO2	85.3 ± 2.5b	90.6 ± 1.1c	85.5 ± 2.5b	90.6 ± 1.1b	70.4 ± 0.3a	73.3 ± 1.1b	39.9 ± 1.3c	39.6 ± 1.1c	61.6 ± 1.6b	54.3 ± 1.5b	42.7 ± 0.7c	31.3 ± 1.2a
NPO3	79.3 ± 3.8c	84.3 ± 2.1d	79.3 ± 3.3c	84.3 ± 2.5c	73.3 ± 0.6b	74.3 ± 1.1b	28.6 ± 1.3d	30.8 ± 0.7d	59.0 ± 2.0b	50.3 ± 0.5c	48.0 ± 0.5d	31.0 ± 0.5a
OPO1	81.0 ± 2.0c	86.6 ± 2.8d	81.0 ± 2.0c	86.6 ± 2.8c	70.3 ± 0.5a	75.3 ± 1.0b	28.9 ± 2.0d	36.6 ± 0.6e	56.0 ± 1.7b	51.0 ± 1.0b	39.7 ± 0.5e	24.3 ± 1.0b
OPO2	82.3 ± 2.0b	83.6 ± 1.3d	82.3 ± 2.1c	83.6 ± 3.0c	67.0 ± 1.5c	65.0 ± 1.5c	36.6 ± 1.6e	36.7 ± 0.4e	59.3 ± 2.1b	51.0 ± 1.0b	34.7 ± 0.5b	24.0 ± 1.0b
OPO3	89.6 ± 3.8b	93.6 ± 2.0b	89.7 ± 3.1b	93.7 ± 2.1b	65.3 ± 1.0c	68.3 ± 1.6c	40.6 ± 1.3f	46.6 ± 1.3b	57.3 ± 1.3b	49.6 ± 1.2b	25.6 ± 0.9f	22.3 ± 1.2b
ONPO1	101.0 ± 2.0d	98.0 ± 2.6c	101.0 ± 2.0d	98.0 ± 2.8d	72.3 ± 0.5b	72.6 ± 0.5b	42.5 ± 2.0c	49.1 ± 0.6f	56.0 ± 1.2b	49.3 ± 2.0b	28.0 ± 1.0a	27.7 ± 0.5c
ONPO2	91.0 ± 2.0e	99.3 ± 1.2c	91.0 ± 2.0b	99.3 ± 2.1d	77.0 ± 1.0d	73.6 ± 1.0b	39.6 ± 2.0f	38.6 ± 0.9f	62.6 ± 0.5b	50.6 ± 1.1b	30.3 ± 0.5a	20.7 ± 1.2d
ONPO3	88.3 ± 1.5b	92.3 ± 1.4b	88.3 ± 2.0b	92.3 ± 1.6b	78.0 ± 0.6d	76.7 ± 1.2d	28.5 ± 2.1d	26.6 ± 1.0e	60.3 ± 1.1b	60.7 ± 1.1d	31.3 ± 0.9a	23.3 ± 0.6b

*Data are means of the triplicate readings (n = 3). Different letters (a–f) in the same column/week represent significant difference at p < 0.05 (Tuckey test). HDL, high density lipoproteins; LDL, low density lipoproteins; ALT, alanine aminotransferase. The 7 and 14 represents the treatment duration*.

Serum glucose level was lower in the un-oxidized PSO supplemented rabbits and decline significantly (*p* < 0.05) further by the supplementation of oxidized PSO. The co-administration increased the levels of serum glucose equals to the levels of un-oxidized PSO fed groups. There was no significant difference during 7 days of treatment in NPO, OPO, and ONPO groups, while significant improvement occurred in the serum glucose in ONPO3 group. Serum ALT levels were higher in PSO fed groups (7 days only) and decreased in oxidized fed groups. Co-administration normalizes the ALT levels.

### Hematological parameters

Blood samples were analyzed for selected important hematological parameters and the results are presented in Table 3. There were no significant (*p* < 0.05) effects on the hemoglobin concentration except in OPO2 group, which showed an increase level. In case of RBC, there was no significant changes with supplementation of un-oxidized pumpkin seed oils alone or in combination with oxidized oil at different doses. Similarly, no significant effects were observed in WBC levels except in OPO3 group, where a significant decrease occurred. Platelets showed a significant decline in OPO3 group only during 7 days of treatment, while no significant difference in any other treatment. The platelets were also declined in NPO2, OPO1, and OPO3 groups, while no significant difference in any other treatment groups during 14 days of treatment.

**Table 3:** Effects of oxidized and un-oxidized pumpkin seed oil supplementation on the selected hematological parameters of rabbits.

**Group**	**Hb**		**RBC**		**WBC**		**Platelets**	
	**7**	**14**	**7**	**14**	**7**	**14**	**7**	**14**
Control	14.2 ± 0.8a	13.9 ± 0.9a	5.98 ± 0.5a	5.91 ± 0.1a	4.14 ± 0.2a	4.90 ± 0.8a	2.90 ± 0.5a	3.11 ± 0.3a
NPO1	11.9 ± 0.2b	12.4 ± 0.8a	6.48 ± 0.1b	6.58 ± 0.9b	3.56 ± 0.1b	5.40 ± 0.9a	3.06 ± 0.4a	2.89 ± 0.4a
NPO2	11.3 ± 0.3b	12.2 ± 0.1a	6.13 ± 0.4b	5.95 ± 0.1a	4.93 ± 0.9c	5.47 ± 0.9a	3.96 ± 0.9a	2.24 ± 0.2b
NPO3	12.7 ± 0.9b	13.2 ± 0.2a	5.86 ± 0.1a	6.50 ± 0.1b	6.56 ± 0.1d	7.00 ± 0.2b	3.84 ± 0.2a	2.85 ± 0.4a
OPO1	12.6 ± 1.5b	12.5 ± 3.3a	5.65 ± 0.3a	6.28 ± 0.2b	5.20 ± 0.2e	5.73 ± 0.6a	2.44 ± 0.9a	2.35 ± 0.8b
OPO2	14.0 ± 0.6a	14.6 ± 1.6a	6.85 ± 0.3b	6.94 ± 0.3b	6.06 ± 0.3f	7.63 ± 0.1b	2.69 ± 0.2a	2.67 ± 0.2a
OPO3	12.9 ± 1.6b	13.5 ± 1.6a	6.60 ± 0.8b	6.43 ± 0.8b	3.96 ± 0.5a	4.76 ± 0.5a	2.06 ± 0.7b	2.38 ± 0.1b
ONPO1	12.0 ± 0.7b	12.5 ± 0.8b	6.37 ± 0.6b	6.45 ± 0.2b	5.23 ± 0.8e	4.00 ± 0.9a	2.81 ± 0.8a	2.81 ± 0.1a
ONPO2	12.9 ± 0.7b	13.8 ± 1.3a	7.05 ± 0.3a	6.82 ± 0.5b	6.66 ± 0.7d	7.73 ± 0.5b	3.46 ± 0.9a	2.84 ± 0.5a
ONPO3	13.9 ± 0.3a	13.4 ± 0.6a	5.95 ± 0.3a	6.28 ± 0.3b	7.50 ± 0.8g	5.13 ± 0.1a	3.24 ± 0.6a	3.03 ± 0.3a

*Data are means of the triplicate readings (n = 3). Different letters (a–g) in the same column/week represent significant difference at p < 0.05 (Tuckey's test). Hb, RBC, WBC represents hemoglobin, red blood cells and white blood cells, respectively*.

### Liver histological studies

**Figure 4:**
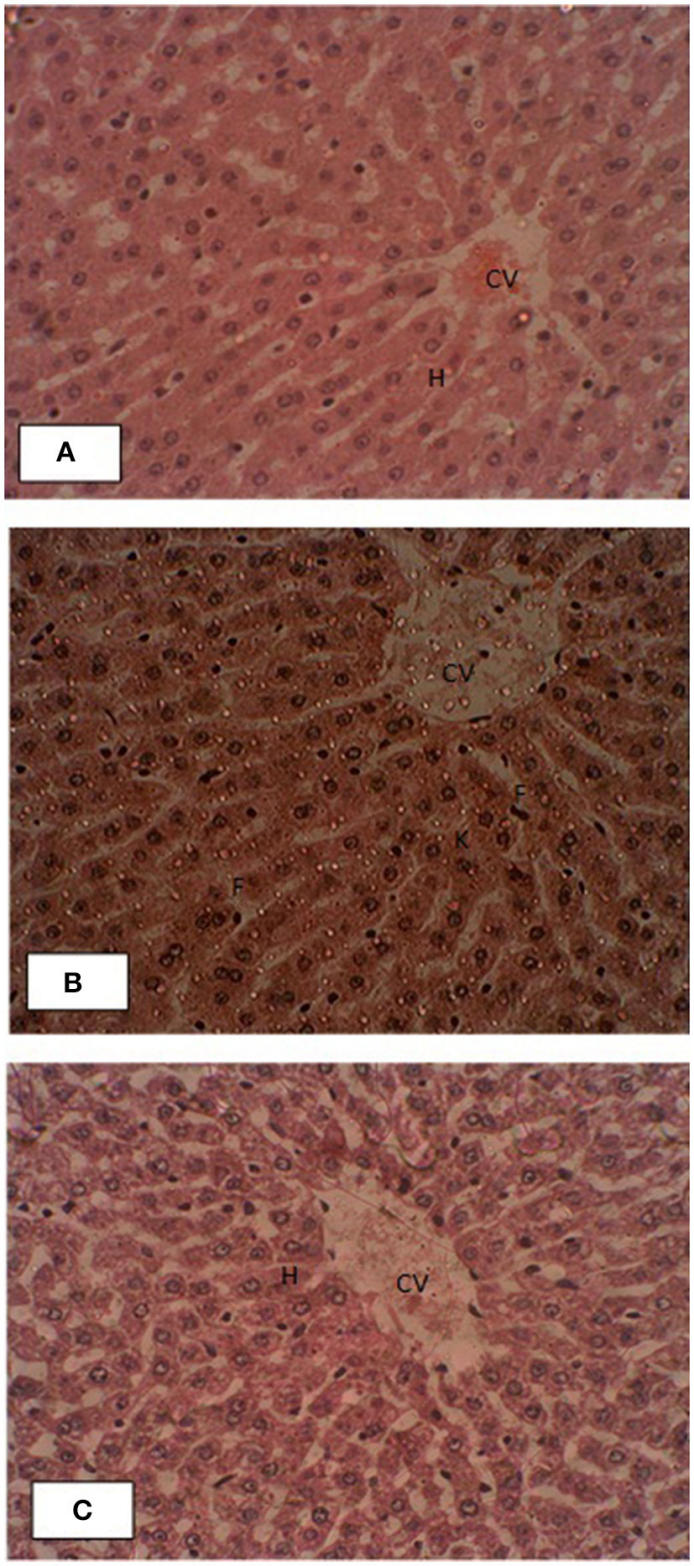
Effects of supplementation of pumpkin seed oil on the liver histology of the rabbit. **(A)** Liver section of control rabbit, **(B)** oxidized pumpkin oil and **(C)** ONPO group. The abbreviations CV, H, F, and K represent central vein, hepatocytes, fats, and reactive Kupffer cells.

Liver histopathology of control and treated groups were performed for effects of pumpkin oil supplementation. Gross morphology of control liver samples showed that the endothelial linings of central veins had normal structure with no evidence of pericentral fibrosis. Kupffer cells are non-reactive. Hepatic cord was very well oriented. The branches of the hepatic artery and portal vein of portal tracts showed normal morphology. No pathological symptoms were present as shown in the Figure 4. All liver slides of treated group showed that the un-oxidized, oxidized pumpkin seed oils alone or in combination had no negative effects on the morphology of the liver.

## Discussion

PSO upon thermal oxidation shows a significant increased in the PV, AV, CD, CT, and TBARS with thermal oxidation. The increase in these quality characteristics was in accordance with the Vujasinovic et al. (2012), who showed that roasting increased the selected quality characteristics of pumpkin seed oil. During thermal edible oils, TAGs are oxidized to primary oxidation compounds such as hydroperoxides, epoxides, and hydroxides (Zeb, 2015). The primary oxidation products are oxidized further to form secondary oxidation products. The primary and secondary oxidation is measured with the help of the above parameters. The increase in the values of these parameters confirms the oxidation of pumpkin seed oil. The increase in the CD and CT values of the pumpkin oils was in accordance with Bardaa et al. (2016), who also showed a similar increase in pumpkin seed oils upon oxidation. The phenolic contents in the studied oil were higher than reported by Andjelkovic et al. (2010). The difference may be due to the difference in the variety, climate and method of analyses. The TPC in PSO decreased with thermal oxidation. The decrease may be due to the degradation of the phenolic compounds (Zeb and Rahman, 2017) in the pumpkin oil.

PSO consists of high amounts of acylglycerols, which includes tri-acylglycerols, mono-acylglycerols (MAGs), di-acylglycerols (DAGs) and oxidized TAGs (TAGOX). Ten triacylglycerols were identified as LLLn, LLL, OLL, OOO, POO, POP, SOO, SSP, SOS, and SSS. The results of TAGs composition are in line with Nederal et al. (2012), who showed that PSO contains LLL, OLL, PLL, LOO, SLL, PLO, PLP, OOO, SOL, POO, POP, and SOO as major TAGs. Unsaturated TAGs, showed a significant decrease in the upon heat treatment. Roasting significantly decreased the amount of ECN 54 TAGs as compared to other TAGs in pumpkin seed oils as indicated by Yoshida et al. (2006). The increase in the amount of saturated TAGs (SOO & SOS) with heating may be due to conversion of unsaturated TAGs to saturated one. This is reflected by a decrease in the iodine values during frying process (Tyagi and Vasishtha, 1996). Comparing the present results with other edible oils under similar conditions of oxidation, we can suggest that PSO from Pakistan was stable for longer duration of heating and could be used as potential frying medium in food industries as well as restaurants.

Supplementation of un-oxidized PSO significantly (*p* < 0.05) increased in the body weight of the rabbits. An increased in the amount of oxidized oil produced significant (*p* < 0.05) increased the body weight as compared to the control. This is in accordance with a recent study, which showed that thermally oxidized rapeseed oil decrease the body weight of the rabbits (Zeb and Rahman, 2017). The co-administration of oxidized oil with un-oxidized significantly increased the body weight. It was thus, concluded that co-administration of un-oxidized PSO with oxidized oil was beneficial in improving body weight. The PSO is usually rich in important phytochemicals i.e., tocopherols and phenolic compounds such as tyrosol, vanillin, vanillic acid, luteolin, and sinapic acid (Andjelkovic et al., 2010), therefore the co-administration helps in increasing the levels of antioxidants and thus was beneficial.

The supplementation of un-oxidized PSO alone increased the levels of TC, TG, HDL, and LDL compared to control, which were declined with an increase in the dose amounts. These results are in agreement with previous study, which showed that TC, TGs, HDL-c, and LDL-c were higher in PSO fed rats (Makni et al., 2008; Gossell-Williams et al., 2011). A significant increase was observed by the supplementation of oxidized pumpkin seed oil, and its effects were minimized by co-administration. In humans, HDL-c levels were improved by supplementation of PSO (Gossell-Williams et al., 2011). This suggests that PSO is beneficial for improving the lipid profile against several stresses such as oxidative stress produced by oxidized lipids.

Serum glucose level was lower in the un-oxidized PSO supplemented rabbits. The reduction in serum glucose may be due to the presence of polyphenolic compounds in pumpkin oil, which improves insulin signaling and lowers the blood HbA1c levels and ultimately reduces serum glucose (Wang and Zhu, 2016). The co-administration of un-oxidized oil with oxidized oils improves the serum glucose level in ONPO3 group. Pumpkin seed had been found to be helpful in prevention of diabetic induced complications (Makni et al., 2008, 2011). These results suggest that pumpkin seed or its oil has hypo-glycemic properties alone, while improving the level in case of oxidized PSO fed groups. Serum ALT levels were higher in PSO fed groups and was lower in oxidized fed groups. Co-administration normalizes the ALT levels. Previous studies showed that supplementation of pumpkin seed extract had hepato-protective role in rats (Makni et al., 2008). This suggests that PSO obtained from the seeds are beneficial against the toxic properties of oxidized lipids in rabbits and different animals.

Toxicological properties of a supplement is determined in terms of blood hematological parameters (Kannan et al., 2012). Results revealed no significant (*p* < 0.05) effects on the hemoglobin concentration except in OPO2 group, which showed an increase level. In case of RBC, and platelet counts, there was no significant changes with supplementation of un-oxidized pumpkin seed oils alone or in combination with oxidized oil at different doses. Similarly, no significant effects were observed in WBC levels except in OPO3 group, where a significant decrease occurred. Previous studies showed that oxidized lipids significantly affected hematological parameters in rabbits (Zeb and Ullah, 2015). This suggests that thermally oxidized pumpkin seed oils alone or in combination with un-oxidized oils have no significant effects on the hematological parameters and thus was beneficial.

Liver histopathology of control and treated groups were performed for effects of pumpkin oil supplementation. The liver of the control animals showed normal structure with no evidence of pericentral fibrosis. Similarly, in the liver slides of all treated groups showed that the un-oxidized, oxidized pumpkin seed oils alone or in combination had no negative effects on the morphology. Recent studies (Zeb and Haq, 2016) showed that thermally oxidized lipids accumulate fats in the liver and resulted inflammation and necrosis. While the present study revealed that pumpkin seed oils oxidized for 9 h have no significant effects, suggesting beneficial properties. The high amount of tocopherol present in the pumpkin oil (Naziri et al., 2016), which may be considered as playing protective role. The low level of oxidation parameters representing stability of the pumpkin oil may also contribute to the positive effects on the liver. The present study is in accordance with the study of Makni et al. (2008), who showed that PSO had hepato-protective role. From these findings, it was concluded that PSO was considered safe for longer frying systems from home to restaurants and was protective against the toxic effects of oxidized lipids.

## Conclusions

PSO was thermally oxidized and evaluated for quality parameters and changes in the acylglycerols composition and biological effects on feeding to rabbits. The levels of quality parameters such PV, AV, TPC, TBARS, CD, and CT increased significantly. HPLC analyses revealed 10 individual TAGs, cumulative amounts of DAGs, MAGs, and oxidized TAGs. The LLL, OLL, OOO, and SSP were present in higher amounts and decreased with thermal treatment. Animals studies showed that oxidized oil decrease the whole body weight, which was recovered by the co-administration of un-oxidized oils. The levels of serum biochemical parameters were maintained by co-administration of pumpkin seed oils. There were no significant effects of both oxidized and un-oxidized PSO on the hematological and histological parameters of rabbits. These results suggest that oxidation of pumpkin seed oils occurred by thermal treatment, but feeding of thermally oxidized oil in combination with un-oxidized PSO has no significant effects upon ingestion and thus considered as safe.

## Ethics statement

The study was carried out under the approval of Graduate studies committee of the Department of Biotechnology, for the proper care and experimentation of the animals and were finally approved by the advanced study and research board (ASRB) of the University of Malakand in its 18th meeting.

## Author contributions

AZ designed experiments, SA collected samples, AZ analyzed for characteristics, SA performed feeding to animals, AZ performed biochemical analysis, SA analyzed hematology and histology, while AZ wrote the manuscript.

### Conflict of interest statement

The authors declare that the research was conducted in the absence of any commercial or financial relationships
